# Pathophysiology of reversible cerebral vasoconstriction syndrome

**DOI:** 10.1186/s12929-022-00857-4

**Published:** 2022-09-21

**Authors:** Shih-Pin Chen, Shuu-Jiun Wang

**Affiliations:** 1 Department of Neurology, Neurological Institute, Taipei Veterans General Hospital, Taipei, 11217 Taiwan; 2 Institute of Clinical Medicine, National Yang Ming Chiao Tung University, Taipei, Taiwan; 3 Division of Translational Research, Department of Medical Research, Taipei Veterans General Hospital, Taipei, Taiwan; 4 Brain Research Center & School of Medicine, National Yang Ming Chiao Tung University, Taipei, Taiwan

**Keywords:** Reversible cerebral vasoconstriction syndrome, Thunderclap headache, Blood–brain barrier, Neurovascular unit

## Abstract

Reversible cerebral vasoconstriction syndrome (RCVS) is a complex neurovascular disorder being recognized during the past two decades. It is characterized by multiple abrupt severe headaches and widespread cerebral vasoconstrictions, with potential complications such as ischemic stroke, convexity subarachnoid hemorrhage, intracerebral hemorrhage and posterior reversible encephalopathy syndrome. The clinical features, imaging findings, and dynamic disease course have been delineated. However, the pathophysiology of RCVS remains elusive. Recent studies have had substantial progress in elucidating its pathogenesis. It is now believed that dysfunction of cerebral vascular tone and impairment of blood–brain barrier may play key roles in the pathophysiology of RCVS, which explains some of the clinical and radiological manifestations of RCVS. Some other potentially important elements include genetic predisposition, sympathetic overactivity, endothelial dysfunction, and oxidative stress, although the detailed molecular mechanisms are yet to be identified. In this review, we will summarize what have been revealed in the literature and elaborate how these factors could contribute to the pathophysiology of RCVS.

## Introduction

Reversible cerebral vasoconstriction syndrome (RCVS) is a complex neurovascular syndrome characterized by multiple abrupt, severe headaches, namely thunderclap headaches, and diffuse segmental constriction of cerebral arteries [1, 2]. The term RCVS was proposed as a unifying nomenclature in 2007 [3] for varieties of historical libeling’s such as Call–Fleming syndrome [4], thunderclap headache with reversible vasospasm [5, 6], benign angiopathy of the central nervous system (CNS) [7], postpartum angiopathy [8, 9], migrainous vasospasm or migraine angiitis [10], or drug-induced cerebral arteritis or angiopathy [11, 12], depending on whether patients present to specialists in stroke, headache, rheumatology, or obstetrics, etc. Accordingly, RCVS has been seen by physicians across disciplines but only until the past two decades has it been recognized as a distinct clinic-radiological syndrome. Large case series of RCVS have been reported in Asia, Europe and America [2, 13–20]. Yet, the exact epidemiology of RCVS remains unclear. Based on an analysis of the United States Nationwide Inpatient Sample database (2016–2017), > 1000 patients with RCVS are hospitalized each year in the entire population of the United States [21]. The survey of hospital-based headache clinics demonstrated that RCVS accounts for nearly 2% of the headache patients in Taiwan [2, 13, 14]. In patients with young stroke (< 45 years), RCVS constitutes up to 13% of the cases in a multicenter Italian study [18]. Hence, RCVS may not be as rare as we thought before. However, whether there is a geographic discrepancy in the prevalence or incidence of RCVS is unclear. Research on this distinct syndrome has accumulated rapidly in an exponential way after the term RCVS being proposed. The clinical features of RCVS have been well delineated; however, the pathogenesis of RCVS remains elusive. Nevertheless, there is substantial breakthrough in understanding the pathogenesis of RCVS during the past few years. In this review, we will briefly outline the clinical features, significance and impact of RCVS and elaborate in detail what has been known on its pathogenic mechanism.

## Clinical features, significance and impact

**Fig. 1:**
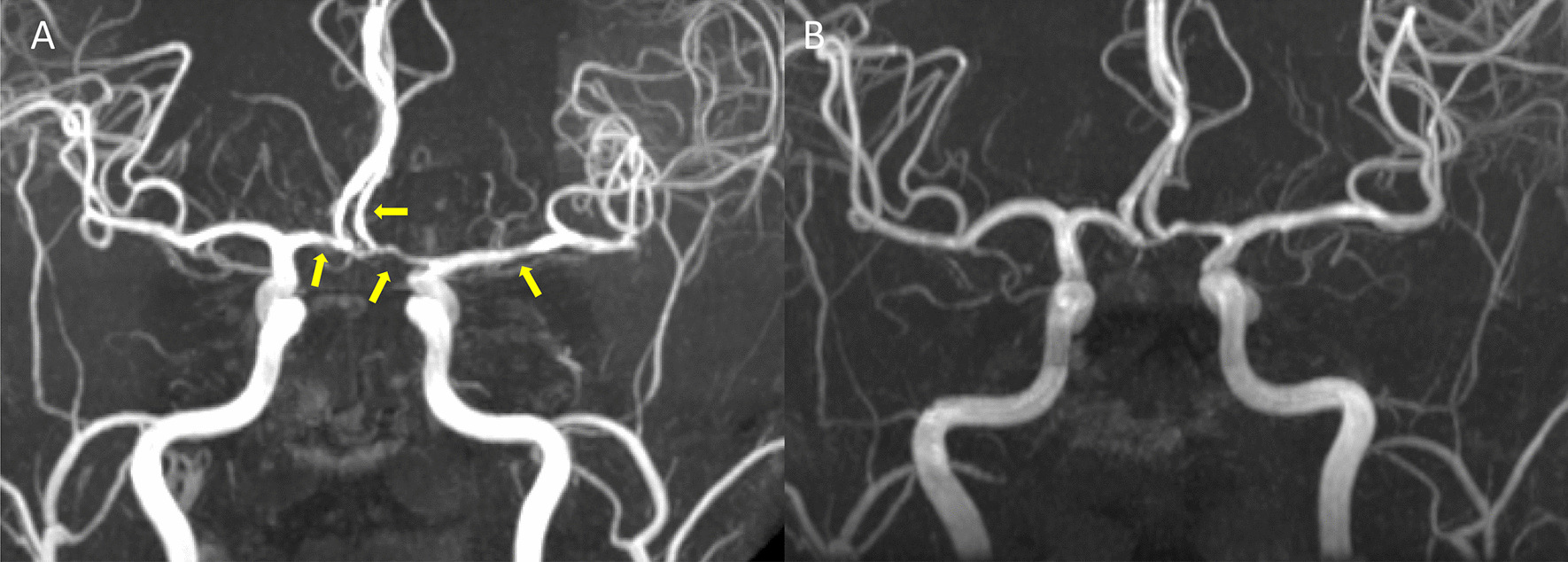
Typical presentations of **A** vasoconstriction and **B** its reversibility in RCVS. Yellow arrow heads indicate segmental vasoconstrictions of major cerebral arteries

RCVS can be either idiopathic [2, 13] or secondary to various factors [15]. An expanding list of possible etiologies of RCVS has been identified [2, 3, 15, 17, 22]. Comprehensive reviews can be found elsewhere [1, 23, 24]. Overall, vasoactive substances and post-partum state are the most common secondary causes of RCVS. In previously reported large series, RCVS is predominately idiopathic in Asian populations while the opposite is observed in Europeans or Americans [2, 13–20]. Patients with RCVS are predominantly female with age around 40–55, while male patients are 10 years younger than the female patients [2, 15, 17]. The clinical hallmark of RCVS, i.e., recurrent thunderclap headaches, are usually evoked by precipitants that are common in daily lives such as defecation, exertion, sexual activity, urination, or cough, bathing or sudden emotional outbursts. Patients often avoid encountering these common daily activities to prevent themselves from the terrifying headaches. These headaches may last for hours long per attack and recur for multiple times within 2–3 weeks of disease onset [2, 15, 17, 25]. Abrupt blood pressure surges accompanying headache attacks can be seen in more than one-third of patients [2, 14, 15]. The vasoconstrictions, which are usually segmental and involving multiple intracranial arteries, may take 3 months to recover (Fig. 1) [13, 14]. The vasoconstriction may sometimes alternate with vasodilatation, involving distal branches of cerebral arteries or arterioles, and propagate centripetally during the disease course [14, 26, 27].

**Fig. 2:**
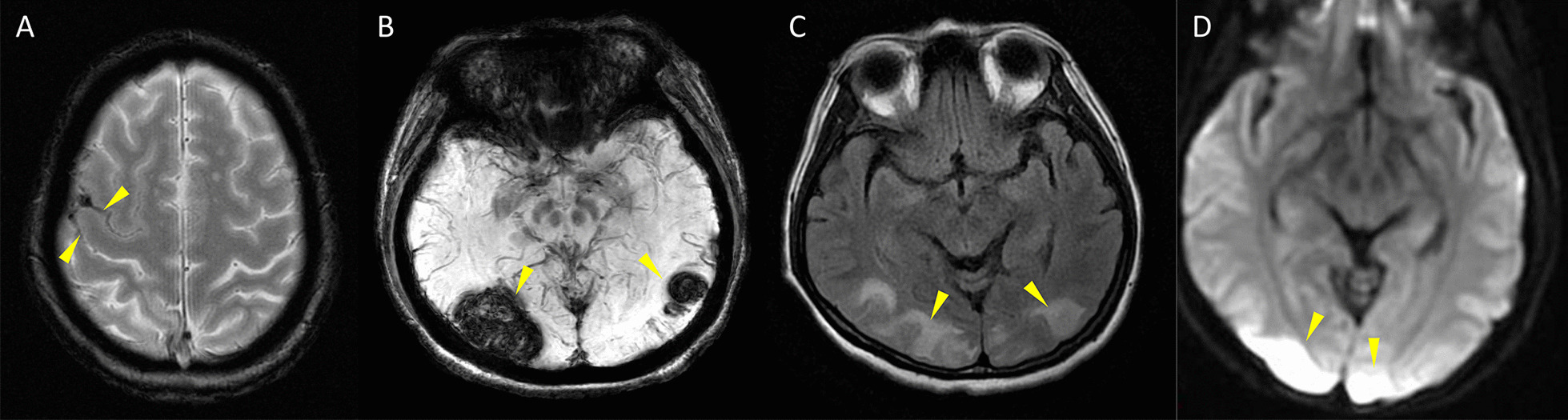
Potential Complications of RCVS. **A** Convexity subarachnoid hemorrhage. The linear hypointensity demonstrated by gradient echo imaging indicated presence of blood within the cortical sulci. **B** Intracerebral hemorrhage shown as hypointense lesions on susceptibility weighted imaging. **C** Posterior reversible encephalopathy syndrome appearing as hyperintense lesions on fluid attenuated inversion recovery imaging. **D** Ischemic stroke demonstrated by diffusion weighted imaging. All the lesions are indicated by yellow arrow heads. Note that **A**–**C** are earlier complications that tend to occur within the first 2 weeks of disease while **D** occurs later at around 2–3 weeks after onset

Not only the severe headache that profoundly interfering quality of life, patients with RCVS may also suffer from potentially devastating complications or comorbid conditions. Nearly one-third of patients with RCVS may experience seizure [2, 3, 15, 17], transient neurological deficits [2, 3, 15, 17], ischemic stroke [13–15, 17, 19], convexity subarachnoid hemorrhage (cSAH) and/or intracerebral hemorrhage [28, 29], or posterior reversible encephalopathy syndrome (PRES) [13–15, 17, 19] during the disease course (Fig. 2). Cervical artery dissection is also a common comorbid condition of RCVS [30]. The severity of vasoconstriction is associated with risks of PRES or ischemic stroke [13, 29] whereas female gender, migraine, history of hypertension, and use of cocaine are risk factors for hemorrhagic complications [16, 29, 31]. Patients may be left with permanent neurological deficits [14, 16, 17, 32]. Death has also been reported in a few cases [17, 33, 34]. Calcium channel blockers, particular nimodipine, may be helpful for aborting headaches [2, 14, 35]. Nearly half of the patients with RCVS may develop post-RCVS headache that could linger for more than 1 year after remission of vasoconstriction [36]. Moreover, 5–10% of the cases may recur during long-term follow-up [37, 38]. Hence, even when the disease has remitted, the long-term risk of RCVS recurrence should not be neglected.

## Pathophysiology

The exact pathophysiology of RCVS remains enigmatic. Because the etiologies of RCVS are heterogeneous, the underlying mechanisms are likely multi-factorial. Observations from clinical features or secondary RCVS have provided important clues to formulate hypotheses. For example, biological effects exerted by the exposed substances [39] or humoral factors associated with puerperium [40, 41] may also contribute to the pathogenesis in idiopathic cases. However, in-depth investigations to validate these hypotheses are relatively scarce. One major limitation is that it is difficult to obtain histopathological samples from the affected vessels or brain. Moreover, in a retrospective study that had 17% of the patients received open brain biopsy or full autopsy [17], extensive histological studies demonstrated no evidence of arterial inflammation or infection. One case report which detailed both histological and electron-microscopical examinations showed that major cerebral arteries were normal except for a patch of subendothelial thickening in the posterior cerebral artery [42]. That is, even with histopathological specimens, no specific pathology has been identified in RCVS. Nevertheless, recent studies have identified some plausible elements that may regulate or reflect the pathogenic alterations of RCVS. Herein we summarized evidence supporting these elements and elaborate how these factors could interact with each other and contribute to the pathogenesis of RCVS.

### Dysregulation of cerebral vascular tone

Based on the radiological features, dysregulation of cerebral vascular tone has been considered the central element in the pathogenesis of RCVS [1, 23, 24]. An abrupt, unpredictable alteration of central vascular tone associated with excessive sympathetic discharge may explain the cerebral vasoconstriction and, the less mentioned, vasodilatation. As pial cerebral arteries are densely innervated by the superior cervical ganglion (and also sparsely by the sphenopalatine, otic or trigeminal ganglion) [43], the release of norepinephrine or neuropeptide Y from the sympathetic nerve endings may cause vasoconstrictions. However, it is uncertain whether the vasoconstriction is the direct consequence of the suddenly increased sympathetic drive caused by triggers or secondary etiologies, or the passive autoregulatory response of the perivascular sympathetic innervation to protect the brain against the abrupt blood pressure surge accompanying the severe headache. Clinical observations support the possibility of both scenarios.

In contrast to pial cerebral arteries, cerebral microcirculation receives intrinsic innervations from the locus coeruleus, raphe nucleus, basal forebrain, or local cortical interneurons [43]. These perivascular nerves intricately regulate the cerebral microvascular tone and autoregulation via populations of receptors within the different cellular compartments of the neurovascular unit. The vascular response of pial cerebral arteries or parenchymal microvasculature to neurotransmitters may differ depending on the distribution of the postjunctional receptors. For instance, norepinephrine causes contraction in the middle cerebral arteries through activation of the α1-adrenoreceptors [44, 45] but elicits dilation of parenchymal arterioles due to the predominant distribution of β-adrenoreceptors [46]. The vasomotor responses of the cerebral microvasculature may be altered preceding the grossly perceivable caliber change of large cerebral arteries, based on the observations of centripetal propagation of vasoconstriction [26, 27] and the different time course of hemorrhagic and ischemic complications [15]. The hemorrhagic complications or brain edema due to the dysfunction of blood–brain barrier (BBB) or neurovascular unit tend to occur during the first 2 weeks of the disease while ischemic complications tend to occur later, either caused by the hypoperfusion due to severe vasoconstriction of major arterial segments or the transformation of vasogenic edema into cytotoxic edema in patients with PRES [1, 6, 14, 23]. In fact, autoregulation failure has been considered important in the pathogenesis of PRES [47, 48], an overlapping syndrome or complication of RCVS.

Direct evidence supporting dysregulation of cerebral vascular tone derived from studies investigating the cerebrovascular reactivity in patients with RCVS [49, 50]. In a retrospective case series, the Breath Holding Index, which measures cerebral endothelium-dependent vasodilation in response to hypercapnia, was severely impaired in patients with RCVS during the acute stage of disease [49]. Similar findings were also observed in another case-control study, which demonstrated not only impaired cerebrovascular reactivity during the acute stage but recovery of the impaired cerebrovascular reactivity in the remission stage in 70% of the cases who received follow-up [50]. Autonomic dysregulation [51], oxidative stress [52, 53], impaired endothelial repairing capacity [54], and disruption of the BBB [55, 56] might contribute to the altered cerebrovascular reactivity, which are detailed below. Moreover, a recent study identified a panel of microRNAs (miR-130a-3p, miR-130b-3p, let-7a-5p, let-7b-5p and let-7f-5p) that can be used to differentiate patients with RCVS (in acute stage) from controls with nearly 90% accuracy [57]. The common targets of these circulating microRNAs include genes responsible for cerebral vascular tone, such as *EDN1* (endothelin-1) or genes involved in the transforming growth factor-beta (TGF-β) signaling pathway [57]. These findings may serve as primers for future studies to investigate potential molecular mechanisms.

### Sympathetic overactivity

Aberrant sympathetic response of cerebral vasculature has been long proposed important in the pathogenesis of RCVS [58], which can be partly supported by the fact that RCVS can occur in some patients with pheochromocytoma [59–61], after the use of sympathomimetic vasoactive substances [12, 14, 15, 17, 62], or acute hypertensive crises [63]. Besides, clinical features such as abrupt blood pressure surge [2, 15] and/or the Valsalva maneuver-like triggers with elevated sympathetic tone [2, 15] also implicate the role of sympathetic overactivity in the pathogenesis.

In a 24-h heart rate variability study, patients with RCVS were found to have heightened sympathetic activity and attenuated parasympathetic modulations during the acute stage [51]. This autonomic dysregulation only partially recovered at the remission stage, suggesting that patients with RCVS might have some predisposition of sympatho-vagal imbalance that makes them vulnerable to external triggers. Alternative explanation is that, once afflicted by the disease, the autonomic dysfunction could not recover soon even after resolution of vasoconstrictions. An indirect support for the sympatho-vagal imbalance predisposition is the higher occurrence of RCVS in cold weather [64]. The sympathetic nervous system might be more excitable in cold weather and thus aggravate a pre-existing sympatho-vagal imbalance and thus induce cerebral vasoconstriction. It has been shown that cerebrovascular resistance increases during cold face stimulation [65] and sympathetic stimulation during cold pressor testing may produce constriction of large cerebral arteries to protect the brain from arterial pressure elevations [66], which might be related mechanistically to cerebral vasoconstriction in RCVS. Additionally, a genetic polymorphism study showed that the *brain-derived neurotropic factor* (*BDNF*) *Val66Met* functional polymorphism is linked to vasoconstriction in patients with RCVS [67]. Preclinical study identified that BDNF could cause perivascular inflammation and vasoconstriction under circumstances of sympathetic overactivity [68]. BDNF could also upregulate neuropeptide Y, a vasoconstrictor secreted by sympathetic nerve endings [69]. Nevertheless, these links are tangential and require further studies to validate.

### Endothelial dysfunction

The cerebrovascular endothelium exerts a profound influence on cerebral vascular tone [70]. It is plausible that the dysregulated cerebral vascular tone in RCVS may in part attributed to endothelium-dependent mechanism. In fact, the impaired endothelium-dependent vasodilation to hypercapnia in patients with RCVS [49, 50] supports this notion. In addition, one study identified that patients with RCVS have reduced circulating CD34^+^KDR^+^ endothelial progenitor cells (EPCs) in comparison with controls, especially in those with more severe vasoconstrictions [54]. The number of CD34^+^KDR^+^ EPCs was negatively correlated with the severity of vasoconstrictions. Because cells expressing CD34 (an adhesion molecule expressed on hematopoietic stem cells) and KDR (a type 2 vascular endothelial growth factor receptor that indicates early endothelial differentiation) might be the main constituent of the circulating EPCs responsible for re-endothelialization [71], it is plausible that patients with RCVS may have reduce impaired endothelium repairing capacity during the acute stage of the disease, either due to lower baseline level or an accelerated consumption and/or senescence of circulating EPCs under the circumstance of sympathetic overactivity and excessive oxidative stress.

Studies from overlapping syndromes or spectral disorders of RCVS also support the existence of endothelial dysfunction at least in selected cases. PRES, a well-recognized cerebral endotheliopathy [72], can be found in a substantial proportion of patients with RCVS [6, 13, 14, 16, 17], and with similar characteristics of RCVS including dysregulated vascular tone, acute severe headaches, blood pressure surge, and secondary etiologies such as immunosuppressive or cytotoxic agents [24, 47, 48]. Post-partum angiopathy, one of the secondary RCVS, exhibits overlapping clinical, laboratory and radiographical features with the obstetric emergencies eclampsia and preeclampsia [22, 73, 74]. Previous studies demonstrated that placental growth factor (PlGF), soluble PlGF receptor (sFlt-1) [40, 41], and soluble TGF-β1 receptor (soluble endoglin) [75] correlate with the presence of eclampsia and the ratio of sFlt-1 to PIGF could also be used to predict the occurrence of pre-eclampsia [41]. The balance of these antiangiogenic and proangiogenic factors could also play a role in the pathogenesis of post-partum angiopathy [42]. Whether patients who are not pregnant or in the puerperium could exhibit similar mechanisms associated with the imbalance of certain humoral factors regulating endothelial function requires further studies.

It has been questioned whether the endothelial dysfunction is restricted to the brain or could be systemic. In a study in which 18 patients with RCVS received transthoracic echocardiography during the acute stage, three (17%) had transient wall motion abnormalities [76]. Another two case reports also demonstrated transient myocardial ischemia [77] or coronary vasospasm [78] responsive to calcium channel blockers. Because of the rarity of these cases, whether RCVS has extracranial vascular involvement remains inconclusive.

### Excessive oxidative stress

Oxidative stress has complex interactions with endothelial dysfunction or sympathetic overactivity to regulate vascular tone [79–82]. Although the idea that excessive oxidative stress may take parts in the pathogenesis of RCVS is intuitive, relevant studies are scarce. A prospective study using liquid chromatography and tandem mass spectrometry (LC-MS/MS) identified that the urine level of 8-iso-prostaglandin F_2α_ (8-iso-PGF_2α_), one of the most reliable oxidative stress markers [83], was elevated in patients with RCVS during the acute stage and normalized during the remission stage [52]. Moreover, 8-iso-PGF_2α_, as a non-enzymatic free radical peroxidation product of arachidonic acid, is also a potent vasoconstrictor [84]. The urine level of 8-iso-PGF_2α_ was found to correlate with the severity of vasoconstriction, especially during the first week of disease course [52]. Consistent with the findings from urine samples, a recent study also demonstrated higher 8-*iso*-PGF_2α_ in the plasma in patients with RCVS during the acute stage [53].

Beyond hypothesis-driven approach, a study combined ^1^H-nuclear magnetic resonance and LC-MS/MS to discover potential urine metabolomic signatures in patients with RCVS [53]. Six metabolites, hippurate, citrate, 1,3,7-trimethyluric acid, ascorbic acid, d-glucurono-6,3-lactone, and d-*threo*-isocitric acid, were identified as the most discriminative metabolites for RCVS. Of them, hippurate, citrate, ascorbic acid, and d-glucurono-6,3-lactone were significantly lower, and 1,3,7-trimethyluric acid and d-*threo*-isocitric acid were higher in RCVS patients. These metabolites are related to two metabolic networks including free radical scavenging and vitamin/mineral metabolism, further validating the potential role of oxidative stress in the pathogenesis of RCVS. Moreover, the hub molecules within the metabolic networks are also associated with endothelial dysfunction or sympathetic overactivity, supporting the complex interaction of these elements in the disease pathogenesis [53].

### Blood–brain barrier disruption

BBB is the main anatomical barrier that separates blood from the microenvironment of brain, regulating the exchange of ions, nutrients, and energy metabolites [85, 86]. The core element of the BBB, the endothelial cells, limits the paracellular and transcellular transport by continuous inter-endothelial tight junctions and the extremely low transcytotic activity. The endothelial cells, pericytes and smooth muscle cells of the vessel, glia cells, and neurons collectively form the neurovascular unit which is responsible for the highly coordinated neurovascular coupling. As vascular tone is delicately regulated by neurovascular unit, it is rational to speculate that a dysfunctional BBB may participate in the pathogenesis of RCVS. When the cerebral autoregulation is profoundly overwhelmed, the integrity of BBB might be breached, and ischemic or hemorrhagic complication might ensue [23, 87]. In fact, reactive oxygen species have been found to contribute to BBB disruption by oxidative damage to cellular molecules, activation of matrix metalloproteinases, reorganization of cytoskeleton, modulation of tight junction proteins, and upregulation of inflammatory mediators [88]. A recent study on the white matter hyperintensity lesions (WMHs) in RCVS also implicates that transmission of excessive central pulsatile flow to cerebral microcirculation upon episodic blood pressure surge may lead to increased microvascular damage, increased vascular permeability, and impaired solute clearance, contributing to WMH formation [89]. With severe breakdown of BBB, hemorrhagic complications or brain edema may ensue.

In 2017, a study using contrast-enhanced fluid-attenuated inversion recovery magnetic resonance imaging (CE-FLAIR) for the first time demonstrated evidence of BBB breakdown in RCVS [55]. This study revealed that nearly 70% of patients with definite RCVS and one-fourth of patients with probable RCVS had BBB breakdown. In addition, BBB breakdown was found to be an independent risk factor for neurological complications, in particular convexity SAH and PRES [55]. A subsequent international collaborative study demonstrates that the BBB breakdown was worse during the first 1–2 weeks and with a temporal course similar to but earlier than that of vasoconstrictions which peaked at the 3rd week [56]. This finding may provide a pathophysiologic background of the temporal course of neurological complications of RCVS, i.e., the hemorrhagic complications or PRES tend to occur early in the disease course whereas ischemic stroke occurs later. In addition, blood pressure surge was found to be a risk factor of BBB breakdown, supporting the speculation of the injurious effect of excessive central pulsatile flow on the brain [89].

**Fig. 3:**
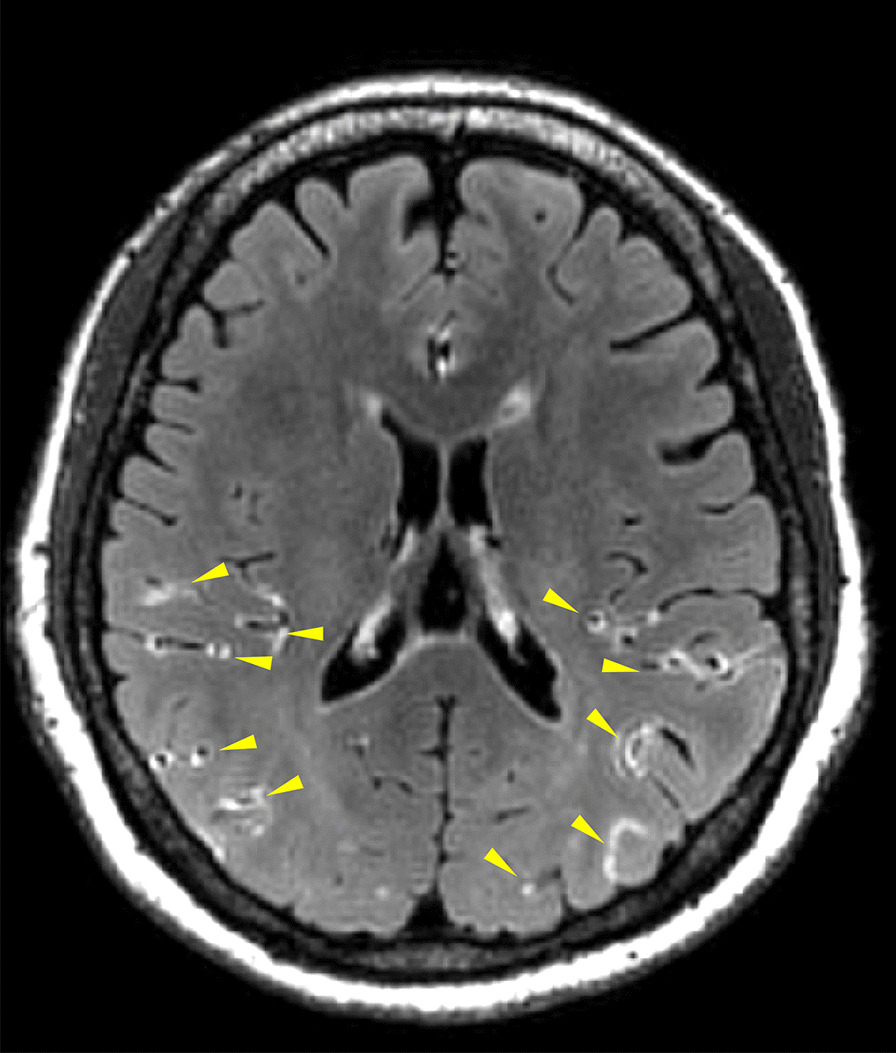
Typical imaging finding of blood–brain barrier breakdown demonstrated by contrast-enhanced FLAIR imaging. The gadolinium-based contrast medium extravasated from the cerebral vessels to the cortical sulci appears hyperintense on FLAIR imaging (yellow arrow heads), providing macroscopic imaging evidence of BBB breakdown

Because the presence of macroscopic BBB disruption in patients with RCVS using CE-T2-FLAIR imaging (Fig. 3) could only be identified in half of the patients with RCVS [56, 90], a recent study interrogated whether the brain permeability in patients without discernible macroscopic BBB disruption is altered using a technique called dynamic contrast-enhanced magnetic resonance imaging (DCE-MRI). The microscopic BBB permeability in the whole brain and WMHs was determined using an index K^trans^ [91]. It was found that patients with RCVS presented increased microscopic permeability of the whole brain and WMHs during acute stage, even without discernible macroscopic BBB disruption on CE-FLAIR. In addition, a dynamic change in BBB permeability was noted, which was correlated with impaired cerebral microvascular compliance assessed with hemodynamic studies and may thus contribute to WMH formation [91].

A recent study identified a panel of circulating microRNAs that may serve as potential disease signature of RCVS [57]. These microRNAs are functionally associated with acute headache, vasomotor control or BBB regulation. Remarkably, miR-130a-3p was associated with imaging-proven BBB disruption in patients with RCVS and its overexpression led to reduced transendothelial electrical resistance (i.e., increased permeability) in in vitro human BBB model [57]. Consistent with this finding, two previous studies have disclosed that miR-130a could increase BBB permeability under ischemic [92] or hemorrhagic conditions [93]. Interestingly, the abundance of miR-130a and miR-130b was associated with blood pressure surge during the acute stage of RCVS. This might be compatible with the mechanosensitive nature of the miR-130/301 family [94], i.e., the expression of miR-130 was upregulated in response to the shearing stress induced by abrupt hemodynamic change upon blood pressure surge. Future studies are needed to explore the mechanism how the upregulated miR-130a interferes BBB permeability.

### Altered trigeminovascular nociception

The cause of thunderclap headache may be the most enigmatic part in the pathogenesis of RCVS. Because the headaches usually recur for multiple times during the first 2 weeks after disease onset while the constriction of major cerebral arterial segments peaked at the 3rd week [13, 14, 56], vasoconstriction of the large or medium-sized cerebral arteries is unlikely to be the cause of the headache. Previously, intracranial pain-sensitive structures are believed to be the dura mater and its feeding vessels. However, a recent study based on the mapping in awake craniotomies in patients suggests that the pia and small cerebral vessels are also pain-sensitive [95]. Based on this updated neuroanatomical knowledge as well as the disease temporal course, the involvement of distal arterioles is more likely the primary trigger activating the trigeminovascular nociceptive pathway in RCVS [95]. Physiologically, the trigeminovascular pathway, which provides the unique sensory innervation to brain vessel, acts as a “protective” system that restores vessel tone after vasocontractile stimuli, probably via the potent vasodilatory neuropeptide calcitonin gene-related peptide (CGRP) released from trigeminal nociceptors [43]. In RCVS, this reflex could be exaggerated but yet to be proved. Increased BBB permeability during the early stage of disease course could also be an important contributor to the headache by activating the trigeminal nociceptors via the leaked intravascular components; however, in-depth studies are still needed.

A recent study found that circulating microRNAs including let-7a-5p, let-7b-5p and let-7f-5p were upregulated both in acute stage of RCVS and ictal stage of migraine and returned to normal in the remission stage of RCVS or interictal stage of migraine [57]. Hence, these microRNAs might be associated with acute pain or certain mechanism shared by RCVS and migraine, such as CGRP-dependent trigeminovascular reflex. Previous studies showed that let-7b is highly expressed in and can be released from dorsal root ganglion (DRG) [96]. Inhibition of let-7b attenuated formalin-induced TRPA1 currents in the DRG neurons and spontaneous pain [96], while TRPA1 activation could trigger CGRP release from the trigeminal ganglion and mediate trigeminovascular reflex [97]. In addition, injecting anti-let-7 into the brain reduces opioid antinociceptive tolerance in mice [98]. Whether these mechanisms could explain the pain in RCVS require further investigations.

### Genetic predisposition

Because of the potential of recurrence [37, 38] and the partially-persistent dysfunctional traits during the remission stage [51], it is reasonable to speculate that patients with RCVS might have some genetic predisposition that makes them vulnerable to the disease. However, no obvious inheritance pattern has been identified. As there are multiple environmental factors that could lead to RCVS, it is likely RCVS is a complex disease modulated by multiple genes with small or moderate effect sizes. Yet, no definite susceptible gene for RCVS has been identified. Only two genetic association studies have been conducted in patients with RCVS. One study found that patients carrying the Val allele of *BDNF Val66Met* polymorphism were more likely to have more severe vasoconstrictions than Met homozygotes [67]; however, the genotype frequencies in RCVS patients were not different from that in normal controls. Another study investigated *Ring Finger Protein 213* (*RNF213*), a susceptibility gene of moyamoya disease and intracranial artery stenosis and dissection, in patients with RCVS found no association between the gene and disease [99].

In a case report, a 9-year-old boy with Loeys–Dietz Syndrome caused by a heterozygous *TGFBR2* (encoding for type II TGF-β receptor) mutation was diagnosed with RCVS with PRES [100], suggesting the potential role of TGF-β signaling pathway in the pathogenesis of RCVS. Interestingly, TGF-β signaling pathway is also the most significant pathway identified by pathway enrichment analysis for the predicted target genes of the RCVS-selective circulating microRNAs [57]. TGF-β has been known to induce the vascular endothelial expression of endothelin-1, a predicted common target of all five RCVS-selective microRNAs. In in vitro study, the expression of *EDN1* expression as well as predicted genes within the TGF-β signaling, including the *TGFBR2*, was inhibited by the patients’ cerebrospinal fluid, validating in silico findings. Although it is likely most RCVS patients would not have monogenic mutations involving *TGFBR2* or *EDN1* genes, it would be interestingly to investigate potential functional polymorphisms of these genes in these patients. Considering the complex nature of the disease, non-hypothesis driven genome-wide based approach might be even helpful to identify possible susceptible genes although this would require a large number of patients via international collaborations.

### Sex hormones

Patients with RCVS are predominantly female of middle age, particularly around perimenopause. Postpartum angiopathy also occurs at the stage when hormones fluctuate dramatically. As cerebral vascular tone and BBB permeability are heavily influenced by the effects of estrogen and progesterone mediated signaling pathways [101], it has long been proposed that sex hormones play important roles in the pathogenesis of RCVS. However, direct evidence is lacking. In a few case reports, the occurrence of RCVS was associated with an acute withdrawal or administration of hormones, such as bilateral salpingo-oophorectomy [102], hormonal ovarian stimulation for intrauterine insemination [103], oral contraceptive pills [104], or levonorgestrel-releasing intrauterine system [105]. However, it is difficult to establish causality based on selected case reports. In a large retrospective study that investigated the potential hormonal influences on RCVS, no significant differences between pre- and post-menopausal women, or those with and without hysterectomy were observed [106]. Hence, it remains inconclusive whether hormonal imbalance could trigger or modify the disease course of RCVS.

### Inflammation

Previously, RCVS was considered a benign angiopathy of the CNS, which should be differentiated from primary angiitis of the CNS (PACNS) [107–109]. Limited histopathological data also do not support the presence of arterial wall inflammation in patients with RCVS [32, 109, 110]. However, marked vascular wall enhancement has been noted in a patient with cocaine vasculitis [111], and cocaine has been considered to be an important etiology of RCVS [1, 17, 109]. A study used high-resolution vascular wall imaging demonstrated enhancement of the diseased vessels in almost half patients with idiopathic RCVS, suggesting that there might be subclinical perivascular inflammation in some patients [112]. Furthermore, a study demonstrated five patients with hemorrhagic PACNS were exclusively associated with sympathomimetic drug exposure and presented as thunderclap headache and self-limited clinical course, which substantially overlapped with the clinical presentations of RCVS [113]. Hence, it is possible that RCVS shared certain pathogenic mechanisms of PACNS, and inflammation could contribute to the vasoconstrictions at least in certain patients. Some other case reports also suggested a potential link between inflammation and RCVS. For example, some patients with systemic lupus erythematosus have been reported to have RCVS [114–116]. Coronavirus disease 2019 (COVID-19) and the multisystem inflammatory syndrome in children (MIS-C) following COVID-19, both with remarkable endothelial inflammation, were also recently linked to RCVS [117–122]. Further studies are needed to elucidate the potential causality and mechanisms between these inflammatory disorders and RCVS.

### Proposed model of the pathophysiology of RCVS

**Fig. 4:**
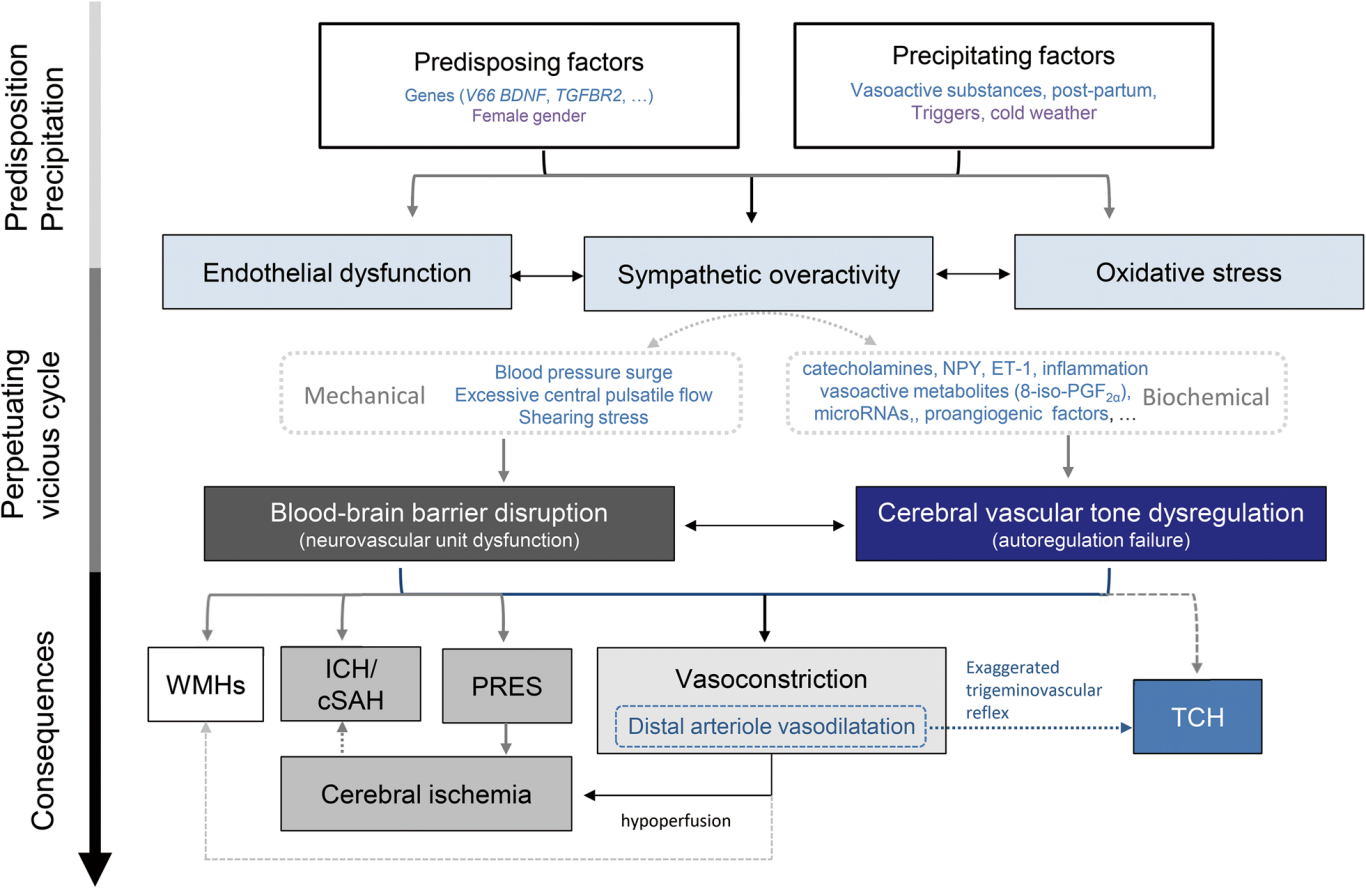
Proposed model of the pathophysiology of RCVS. Development of RCVS is sequential, which may require both predisposition and precipitating factors to initiate and perpetuate a vicious cycle of pathogenic mechanisms that result in the clinical and radiological manifestations (as indicated by the gradient arrow on the left of the figure). Dysregulation of cerebral vascular tone and disruption of blood–brain barrier (BBB) (i.e., dysfunctional neurovascular unit) might play the key roles in the pathophysiology of RCVS, which could be mediated by the mechanical and biochemical consequences of heightened sympathetic drive, endothelial dysfunction and oxidative stress. This could particularly occur in vulnerable subjects with certain predisposition when they encounter secondary causes (e.g., vasoactive substances, post-partum state, etc.), triggers (e.g., exertion, defecation, sexual activity, cough or bathing), or adverse environment (e.g., cold weather). When the dysfunctional autoregulation and BBB disruption exacerbate and the endogenous protective mechanisms fail, headache, vasoconstrictions, and complications may ensue. The thunderclap headache could be attributed either to the dilatation of distal arterioles or meningeal arteries, that activate the trigeminovascular nociceptive fibers. Hemorrhagic complications (cSAH and ICH) or PRES may be attributed to the breakdown of BBB, while the ischemic stroke is related to hypoperfusion caused by vasoconstriction of major cerebral arteries. White matter hyperintensity lesions could be attributed to either increased BBB permeability or partial ischemia due to cerebral hypoperfusion. *BDNF* brain-derived neurotrophic factor, *cSAH* convexity subarachnoid hemorrhage, *ET-1* endothelin-1, *ICH* intracerebral hemorrhage, *NPY* neuropeptide Y, *PRES* posterior reversible encephalopathy syndrome, *TCH* thunderclap headache, *TGFBR2* transforming growth factor-beta receptor 2, *WMH* white matter hyperintensity lesion

Based on the potential elements elaborated above, we propose a model for the pathophysiology of RCVS (Fig. 4) and summarize the main mechanism and strength of evidence for each proposed element of pathophysiology (Table 1). Patients with certain predisposition (possibly related to some susceptible genes) may be more vulnerable to triggers with heightened sympathetic drive, secondary causes (such as the use of vasoactive substances or postpartum), systemic inflammation, or adverse environments such as cold weather. When the central or systemic sympathetic system is ignited and overacted, a sudden release of vasoconstrictors such as catecholamines, neuropeptide Y or endothelin-1 may cause an abrupt dysregulation of cerebral vascular tone. The impaired autoregulation may initially manifest as dilatations of distal arterioles, partly due to an exaggerated trigeminovascular reflex. The dilatation of distal arterioles, capillaries or meningeal collaterals may abruptly stretch the perivascular nociceptive nerve fibers to cause thunderclap headaches. In addition, excessive central pulsatile flow related to blood pressure surge may cause the dysfunction of neurovascular unit and increased BBB permeability, which may also contribute to impaired cerebral vascular tone and headache. Furthermore, to counteract the excessive pulsatile flow and distal arteriole dilation, the large or medium-size cerebral arteries constrict subsequently, manifesting as centripetal propagation of vasoconstrictions during the disease course.

**Table 1:** Summary of potential pathophysiology of RCVS

Key elements	Main mechanism	Strength of evidence	Possible treatment strategies^a^
Dysregulation of cerebral vascular tone	• Suddenly increased sympathetic drive • Passive autoregulatory response against blood pressure surge • Consequences of endothelial dysfunction, oxidative stress, and BBB disruption • Dysregulated circulating miRNAs associated with vasomotor regulation	+++	• Early identification and avoidance of triggers or secondary causes with heightened sympathetic drive • Cerebrovascular-selective calcium channel blockers?
Sympathetic overactivity	• Predisposing sympatho-vagal imbalance • Triggers or secondary causes associated with heightened sympathetic drive	++	• Early identification and avoidance of triggers or secondary causes
Endothelial dysfunction	• Impaired endothelial repairing capacity (i.e., reduced endothelial progenitor cells) • Systemic endotheliopathy caused by secondary causes	+++	• Avoidance or early removal of secondary causes
Excessive oxidative stress	• Increased reactive oxygen species and lipid peroxidation upon increased shearing stress and endothelial dysfunction	++	• Antioxidants?
Blood–brain barrier disruption	• Excessive blood pressure surge and pulsatile flow exceeding autoregulatory capacity • Oxidative stress • Circulating microRNAs (miR-130a)	+++	• Avoid blood pressure surge • Antioxidants?
Altered trigeminovascular nociception	• Sudden stretch of perivascular trigeminal nociceptors (by dilatation of distal arteriole or meningeal artery) • Increased BBB permeability • Altered circulating microRNAs	?	• The cerebrovascular-selective calcium channel blocker nimodipine • CGRP-targeting therapy? (with risk of vasoconstriction)
Genetic predisposition	• Genetic contribution to disease vulnerability	+	• Gene-based mechanism targeting therapy?
Sex hormones	• Hormonal modulation of cerebral vascular tone	+	• Avoid abrupt hormonal fluctuation in vulnerable subjects?
Inflammation	• Systemic or perivascular inflammation causing endothelial dysfunction	+	• Anti-inflammatory therapy? (Of note, steroid may be associated with poor prognosis)

Strength of evidence: +++: supported by multiple studies from different research groups; ++: supported by at least one study from single research group; +: based only on clinical observations; ?: based only on hypothesis

^a^Treatment without direct supporting evidence is ended with a question mark

With persistently heightened sympathetic drive, endothelial dysfunction and excessive production of oxidative stress ensue, leading to a vicious cycle that further perpetuates the dysregulation of cerebral vascular tone. The endogenous endothelial repairing capacity may thus be partially exhausted. The vasoactive metabolites produced in response to free radicals such as 8-iso-PGF_2α_ or the intravascular plasma components leaked to the paravascular CSF space due to BBB disruption may further aggravate vasoconstriction. Inflammation of vascular wall may develop in selected cases along with prolonged vasoconstrictions. In addition, in response to the shearing stress of excessive blood flow and deranged vascular tone, the expression of some microRNAs including miR-130a-3p, miR-130b-3p, let-7a-5p, let-7b-5p and let-7f-5p is upregulated. While countering the effect of vasoconstrictive genes such as *EDN1* or those within the TGF-β signaling pathway, these microRNAs may also differentially participate in the genesis of the acute headache or BBB disruption.

The breakdown of BBB and excessive central pulsatile flow may lead to WMHs as well as the early complications such as PRES, convexity SAH or intracerebral hemorrhage. In contrast, vasoconstrictions of major arteries may lead to hypoperfusion, which might aggravate WMHs or when profound, lead to ischemic complications or aggravate the vasogenic edema of PRES lesions to cytotoxic edema.

Of note, because of the limitation of available studies, only some of the aforementioned mechanisms such as sympathetic overactivity or endothelial dysfunction can be applicable to both idiopathic RCVS and RCVS with secondary causes. As most of the mechanisms with better supporting evidence were derived from studies involving predominantly idiopathic cases, whether these mechanisms could be extrapolated to RCVS with secondary causes require further studies.

## Conclusion

RCVS is a complex neurovascular disorder with dramatic clinical presentation, dynamic disease course, and potentially devastating complications. Recent research has provided substantial progress in understanding its pathophysiology, particularly on the dysregulation of cerebral vascular tone and disruption of BBB, although vast areas of unknowns remain to be explored. By unraveling the complex neurobiology of RCVS, we may be able to subclassify the syndrome with pathophysiology-specific perspectives and formulate potential disease-targeting management in the future.

## Data Availability

All data are available in the manuscript and figures.
